# Factors associated with recurrence and mortality in central line-associated bloodstream infections: a retrospective cohort study

**DOI:** 10.1186/s13054-018-2206-7

**Published:** 2018-10-26

**Authors:** Luis E Huerta, George E Nelson, Thomas G Stewart, Todd W Rice

**Affiliations:** 1 Division of Allergy, Pulmonary, and Critical Care Medicine, Vanderbilt University Medical Center, 1161 21st Ave S., T-1218 MCN, Nashville, 37232-2650 TN USA; 2 Division of Infectious Diseases, Vanderbilt University Medical Center, Nashville, TN USA; 3 Department of Biostatistics, Vanderbilt University Medical Center, Nashville, TN USA

**Keywords:** Catheter-related infections, Cross infection, Anti-infective agents, Critical care

## Abstract

**Background:**

Central line-associated bloodstream infections (CLABSIs) are associated with increased mortality, hospital length of stay, and cost. Antimicrobial treatment guidelines for CLABSIs are primarily based on expert opinion. We hypothesized that shorter antimicrobial treatment duration is associated with decreased 60-day recurrence-free survival.

**Methods:**

A retrospective cohort study of all adults with hospital-acquired CLABSIs (HA-CLABSIs) over 5 years at a single tertiary care academic hospital was performed. The time from the end of effective antimicrobial treatment until recurrence of infection or mortality, censored at 60 days after the end of antimicrobial treatment, represented the primary outcome. Effective antimicrobial treatment was defined as the administration of at least one antimicrobial to which the causative organism was sensitive.

**Results:**

A total of 366 cases met eligibility criteria. The median Sequential Organ Failure Assessment (SOFA) score was 6 (interquartile range (IQR) 4–8). Patients were treated for a median of 15 (IQR 10–20) days with effective antimicrobials. The incidence of 60-day mortality or recurrence after completion of the antimicrobial course was 22.1% (81 patients). In a Cox proportional-hazards model, antimicrobial treatment duration (hazard ratio (HR) = 0.35; 95% confidence interval (CI) 0.26–0.48), SOFA score (HR = 1.16; 95% CI 1.09–1.22), and age (HR = 1.021; 95% CI = 1.004–1.037) were associated with mortality or recurrence. The effect of antimicrobial treatment duration appeared to plateau after 15 days.

**Conclusions:**

Longer antimicrobial treatment duration in patients with HA-CLABSIs is associated with improved recurrence-free survival during the first 60 days after infection. This effect appears to plateau after 15 days of treatment. Prospective studies are needed to definitively determine the optimal antimicrobial treatment duration for CLABSIs.

**Electronic supplementary material:**

The online version of this article (10.1186/s13054-018-2206-7) contains supplementary material, which is available to authorized users.

## Background

Hospital-acquired central line-associated bloodstream infections (HA-CLABSIs) contribute significantly to morbidity and mortality in intensive care units (ICUs). Based on Centers for Disease Control and Prevention (CDC) data from 2014, over 35,000 HA-CLABSIs occur annually, with about half of them in ICUs [1]. CLABSIs have serious clinical and financial ramifications; each individual CLABSI is estimated to cost about $40,000 in the medical ICU [2–4] and is associated with a doubling of the risk of mortality, even after adjusting for severity of illness [5]. Central line infections have also been associated with increased ICU and hospital lengths of stay [2, 4, 6, 7].

Treatment for central line infections consists of antimicrobial therapy and, frequently, removal of the infected central line [8]. While some evidence exists to guide antimicrobial treatment duration in patients with bacteremia, evidence in patients with central line infections is limited [9, 10]. Organism-specific treatment guidelines from the Infectious Diseases Society of America (IDSA) exist, but its antimicrobial treatment duration recommendations are based on expert opinion and a few observational studies [11–13]. The ideal antimicrobial treatment duration to prevent mortality and recurrent infection in patients with HA-CLABSIs remains uncertain. The purpose of this study was to investigate factors related to recurrence and mortality in patients with HA-CLABSIs and to determine if longer antimicrobial treatment duration was associated with improved outcomes. We hypothesized that prolonged duration of antimicrobial treatment would increase time until recurrence or mortality.

## Methods

We performed a retrospective cohort study of adults 18 years or older with HA-CLABSIs at one tertiary care academic medical center with approximately 40,000 annual inpatient admissions over a 5-year period (1 July 2010 through 30 June 2015) to study factors associated with 60-day mortality or recurrent infection. The Vanderbilt University Medical Center Institutional Review Board approved the study with a waiver of informed consent. Portions of this research have previously been presented in abstract form [14].

### Subjects

All adults who developed HA-CLABSIs between July 2010 and June 2015 were included. This data collection period ensured that the same CLABSI treatment guidelines were in effect throughout the entire study period [11]. The cohort of patients was identified via a pre-existing list of prospectively collected HA-CLABSI cases reported to the CDC’s National Healthcare Safety Network (NHSN) and maintained for quality improvement purposes. These cases were originally adjudicated by a multidisciplinary infection prevention team using the CDC definitions of central venous catheters and HA-CLABSIs [15]. Specifically, this definition excluded patients with a documented source of infection other than a central venous catheter (e.g., patients with endocarditis or osteomyelitis and resultant bacteremia with a central line in place at the time of the bacteremia). This case list was further reviewed by the first author to confirm that all cases met the current HA-CLABSI definition [15]. Patients who died within 1 day of their last dose of effective antimicrobials were excluded from the primary analysis to correct for the possibility of survivor bias, in which people who die before completing their antimicrobial treatment might spuriously strengthen an association between mortality and shorter antimicrobial treatment duration. These definitions were finalized prior to data collection and analysis.

### Data collection

Information on demographics, comorbidities, central line characteristics, causative organisms, treatments, and clinical outcomes was extracted from the medical records if not previously collected for quality improvement purposes. Mortality data were collected from the medical record and the Social Security Death Index. Data on comorbidities were electronically captured using ICD-9 codes. The remaining data were abstracted manually by the first author.

Immunosuppression was defined by either infection with human immunodeficiency virus (HIV) with a CD4 count less than 200 cells/mm^3^, an absolute neutrophil count less than 500 cells/mm^3^, or a history of organ transplant with ongoing receipt of at least one immunosuppressant medication. Severity of illness on the date of CLABSI diagnosis was measured using the Sequential Organ Failure Assessment (SOFA) score, with partial pressure of oxygen in arterial blood (PaO_2_) to fraction of inspired oxygen (FiO_2_) ratios estimated by oxygen saturation (SpO_2_) to fraction of inspired oxygen (FiO_2_) ratios if no PaO_2_ to FiO_2_ ratios were available [16–18]. Causative organisms were categorized into those with a presumed higher risk of recurrence (prespecified as *Staphylococcus aureus*, *Pseudomonas* species, and *Candida* species) and those with a presumed lower risk of recurrence (all others). Central line removal was defined as the removal of all causative central lines (as per CDC definitions) within 4 days of CLABSI diagnosis [11]. Patients in whom this did not occur were defined as having persistent central lines. Given that growth and speciation of organisms may take 2–3 days after drawing blood cultures (the date of diagnosis according to CDC definitions), clinicians would generally have had 24–48 h after receiving blood culture results to remove an infected central line for it to be considered removed in this study. Duration of antimicrobial treatment was defined as the total number of days during which the patient received effective antimicrobial treatment on or after the date of CLABSI diagnosis. Effective antimicrobial treatment was defined as the receipt of at least one dose of an antimicrobial to which the causative organism was sensitive, as determined by cultures, or having a therapeutic level of an antimicrobial, if levels were measured (e.g., vancomycin). The final day of antimicrobial therapy was defined as the last day on which the patient received effective antimicrobials (or was known to have therapeutic antimicrobial levels), followed by at least 2 consecutive days without effective antimicrobial treatment. No protocols mandated antimicrobial treatment duration or a specific diagnostic workup. Patient workup and treatment were determined by the treating clinician.

### Outcomes

Time until recurrence of infection or all-cause mortality, censored 60 days after the end of antimicrobial treatment, represented the primary outcome. Data from outpatient records and the Social Security Death Index were utilized to determine recurrence or mortality after hospital discharge. The baseline time was defined as the final day of effective antimicrobial therapy. Secondary outcomes were the composite of 60-day mortality or recurrence, 60-day mortality, and 60-day recurrence, measured from the same baseline time.

Subsequent infection was considered recurrent if the culture from a new infection grew the same species as the initial causative organism (or, in the case of a polymicrobial infection, if the new culture grew one or more of the original causative organisms) regardless of the organisms’ antimicrobial sensitivity profiles. As with the initial infection, when a recurrent infection was diagnosed via blood cultures, two positive cultures were required to diagnose recurrence of common commensal organisms. In addition, an infection was only defined as recurrent if the new culture was obtained after completion of the initial effective antimicrobial course. All outcomes were censored at 60 days after the completion of antimicrobial treatment.

### Sample size

Simulation studies for Cox proportional hazards models suggest that approximately 10–15 outcome events per degree of freedom prevents overfitting [19–21]. Given the fixed sample size of 366 eligible HA-CLABSIs with 81 meeting the primary outcome, we limited covariates, interactions, and nonlinear terms to 8 or fewer degrees of freedom.

### Statistical methods

Categorical variables are described using numbers and percentages. Continuous variables are described using medians and interquartile ranges (IQRs). Univariate analyses were performed using Chi-square or Fisher’s exact tests for categorical variables, and Wilcoxon rank-sum tests for continuous variables. Multivariate analysis of the primary outcome, time to 60-day mortality or recurrence, was performed with a Cox proportional-hazards model. Covariates for the model, prespecified based on clinical judgment, were: age, sex, immunosuppression status, SOFA score, infection with a high-risk organism, removal of all central lines within 4 days of infection, and antimicrobial treatment duration. For antimicrobial treatment duration, nonlinearity was allowed using restricted cubic splines with three knots. The proportional hazards assumption was assessed graphically. Individual Cox proportional-hazards models were also developed to test the secondary outcomes of time to 60-day mortality and time to 60-day recurrence separately. As sensitivity analyses, the composite of 60-day mortality or recurrence and its individual components were also evaluated with logistic regression models. Four separate sensitivity analyses were performed excluding patients with *Staphylococcus aureus*, patients with polymicrobial infections, patients who received no effective antimicrobial therapy, and patients who received fewer than 6 or greater than 16 days of antimicrobials. Another sensitivity analysis was performed among patients whose central lines were removed in which antimicrobial treatment was not defined as effective until after all central lines were removed. To evaluate the stability of findings depending on the selected time zero (the end of effective antimicrobial treatment in the primary analysis), a sensitivity analysis was performed assuming that 14 days of antimicrobial treatment was a “full course” and thus including all outcomes starting 15 days after CLABSI diagnosis, even if patients were receiving effective antimicrobials at the time (making time zero the earlier of either the end of effective antimicrobial treatment or day 14 of antimicrobial treatment). Statistical significance was set at a two-sided *P* value less than 0.05. Patients with missing data were excluded. Statistical analyses were performed with R Version 3.4.1 (R Foundation for Statistical Computing, Vienna, Austria).

## Results

**Fig. 1:**
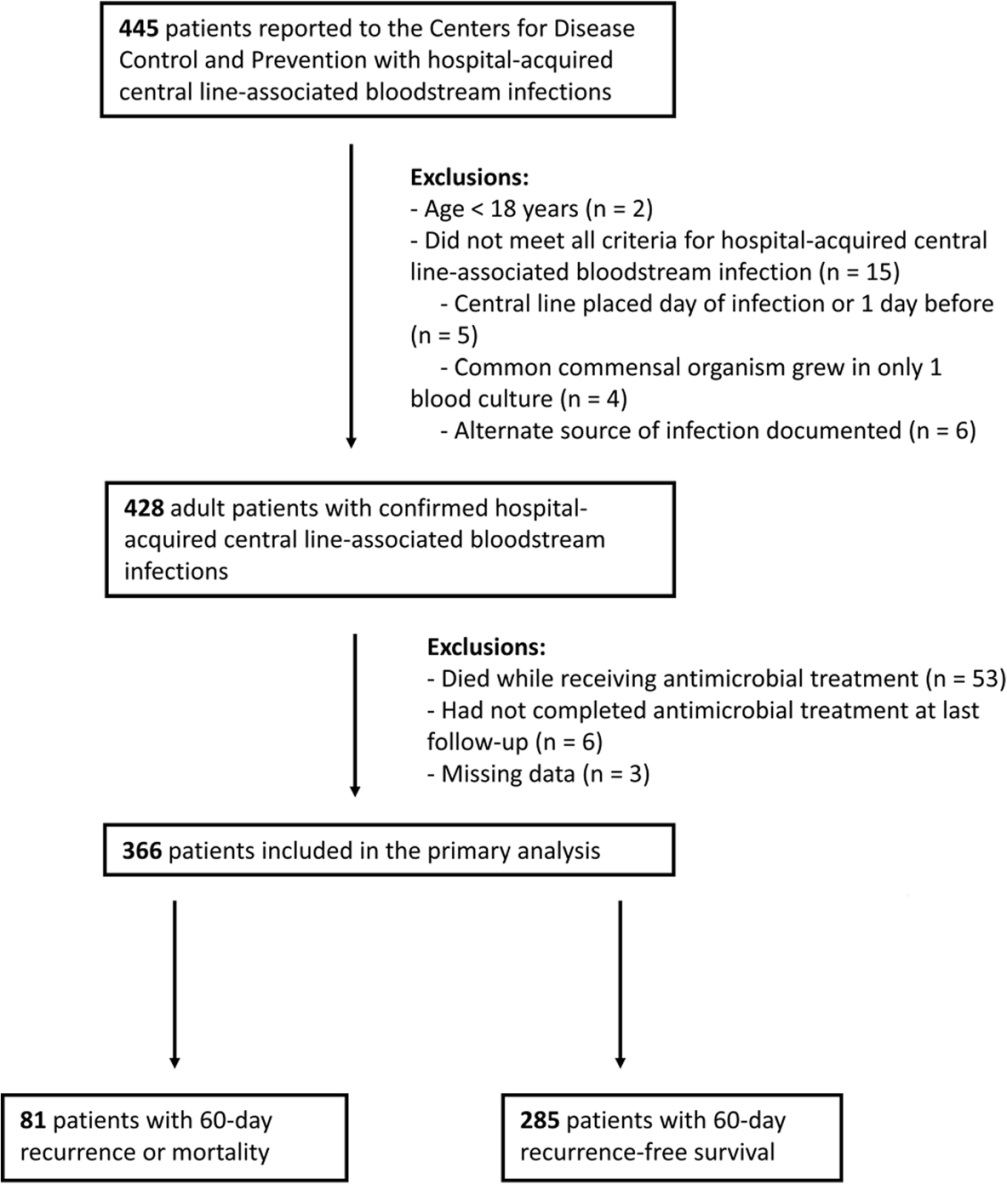
Flow diagram for the study

During the study period, 445 patients had HA-CLABSIs reported to the CDC, of which 366 met eligibility criteria and were included in the primary analysis (Fig. 1). The most common reason for exclusion was death during antimicrobial therapy, occurring in 53 patients.

Baseline characteristics are listed in Table 1. One-hundred and thirty-six patients (37.2%) were immunosuppressed. Patients received a median of 15 days of effective antimicrobials (IQR 10–20), and 272 patients (74.3%) had all central lines removed within 4 days of CLABSI diagnosis, with a median time of 1 day (IQR 0–2) from diagnosis to removal. Of the 49 patients (13.4%) with *Staphylococcus aureus* CLABSIs, 32 (65.3%) had methicillin-resistant *Staphylococcus aureus*. Forty-nine patients (13.4%) had polymicrobial infections. Outcomes data were available for a median of 60 days after completing antimicrobial treatment (IQR 45–60). Central line characteristics are listed in (Additional file 1: Table S1).

**Table 1:** Patient characteristics

Characteristics	Complete cases (*N* = 366)	Patients with 60-day mortality or recurrence (*N* = 81)	Patients with 60-day recurrence-free survival (*N* = 285)	*P* value^a^
Age (years)	55 (40–64)	59 (48–65)	53 (38–63)	0.005
Male	225 (61.5)	48 (59.3)	177 (62.1)	0.64
Hospital length of stay prior to CLABSI (days)	12 (6–18)	12 (6–19)	11 (6–18)	0.70
In ICU at CLABSI diagnosis	119 (32.5)	32 (39.5)	87 (30.5)	0.13
Immunosuppression^b^	136 (37.2)	31 (38.3)	105 (36.8)	0.81
End-stage renal disease	19 (5.2)	7 (8.6)	12 (4.2)	0.15
Cirrhosis	14 (3.8)	7 (8.6)	7 (2.5)	0.02
Diabetes mellitus	106 (29.0)	26 (32.1)	80 (28.1)	0.48
Shock^c^	50 (13.7)	16 (19.8)	34 (11.9)	0.07
Invasive mechanical ventilation^d^	89 (24.3)	23 (28.4)	66 (23.2)	0.33
SOFA score	6 (4–8)	6 (4–9)	5 (3–7)	0.002
Causative organism^e^				
*Staphylococcus aureus*	49 (13.4)	10 (12.4)	39 (13.7)	0.75
Methicillin-resistant *Staphylococcus aureus*	32 (8.7)	7 (8.6)	25 (8.8)	0.97
Coagulase-negative *Staphylococcus*	60 (16.4)	12 (14.8)	48 (16.8)	0.66
*Enterococcus* species	81 (22.1)	19 (23.5)	62 (21.8)	0.74
Gram-negative bacilli	125 (34.2)	25 (30.9)	100 (35.1)	0.48
*Pseudomonas* species	18 (4.9)	5 (6.2)	13 (4.6)	0.56
Other bacteria	49 (13.4)	8 (9.9)	41 (14.4)	0.29
Fungi	41 (11.2)	13 (16.1)	28 (9.8)	0.12
*Candida* species	37 (10.1)	12 (14.8)	25 (8.8)	0.11
Polymicrobial	49 (13.4)	10 (12.4)	39 (13.7)	0.75
High-risk organism^f^	104 (28.4)	27 (33.3)	77 (27.0)	0.27
All central lines removed^g^	272 (74.3)	51 (63.0)	221 (77.5)	0.008
Days until central lines removed^h^	1 (0–2)	1 (0.5–2)	1 (0–2)	0.18
Antimicrobial treatment duration (days)	15 (10–20)	11 (4–18)	15 (12–20)	< 0.001

Values are presented as number (percentage) or median (interquartile range) as appropriate

*CLABSI* central line-associated bloodstream infection, *ICU* intensive care unit, *SOFA* Sequential Organ Failure Assessment

^a^Univariate analyses were performed with Mann-Whitney *U* tests for continuous variables and Chi-square or Fisher’s exact tests for categorical variables

^b^One hundred and twenty patients were neutropenic (absolute neutrophil count less than 500 cells/mm^3^), 26 were organ transplant patients on immunosuppression, and 2 had human immunodeficiency virus infection with a CD4 count less than 200 cells/mm^3^

^c^Defined as the receipt of any vasoactive medication on the date of CLABSI diagnosis

^d^Defined as the receipt of any invasive mechanical ventilation on the date of CLABSI diagnosis

^e^Patients could have multiple causative organisms; numbers may not add up to 100%

^f^Prespecified as *Staphylococcus aureus*, *Pseudomonas* species, and *Candida* species

^g^Defined as patients with all causative central lines removed within 4 days of CLABSI diagnosis

^h^*N* = 272 for complete cases; *n* = 51 for patients with 60-day mortality or recurrence; *n* = 221 for patients with 60-day recurrence-free survival

### Outcomes

Sixty-day mortality or recurrence occurred in 81 patients (22.1%); 26 (7.1%) developed recurrent infection, while 62 (16.9%) died (seven patients died after developing a recurrent infection). Among patients who experienced mortality or recurrence, the median time to event was 10 days (IQR 3–22) after completion of effective antimicrobial therapy. In univariate analyses, shorter antimicrobial treatment duration (*p* <  0.001), increasing SOFA score (*p* = 0.002), increasing age (*p* = 0.005), presence of cirrhosis (*p* = 0.02), presence of a femoral central line (*p* = 0.04), presence of multiple central lines (*p* = 0.04), and persistent central lines (*p* = 0.008) were all associated with increased 60-day mortality or recurrence (Table 1, Additional file 1: Table S1). Among the 136 immunosuppressed patients, 60-day mortality or recurrence occurred in 31 patients (22.8%); 22 (16.2%) died and 13 (9.6%) developed a recurrent infection (four patients died after developing a recurrent infection).

**Fig. 2:**
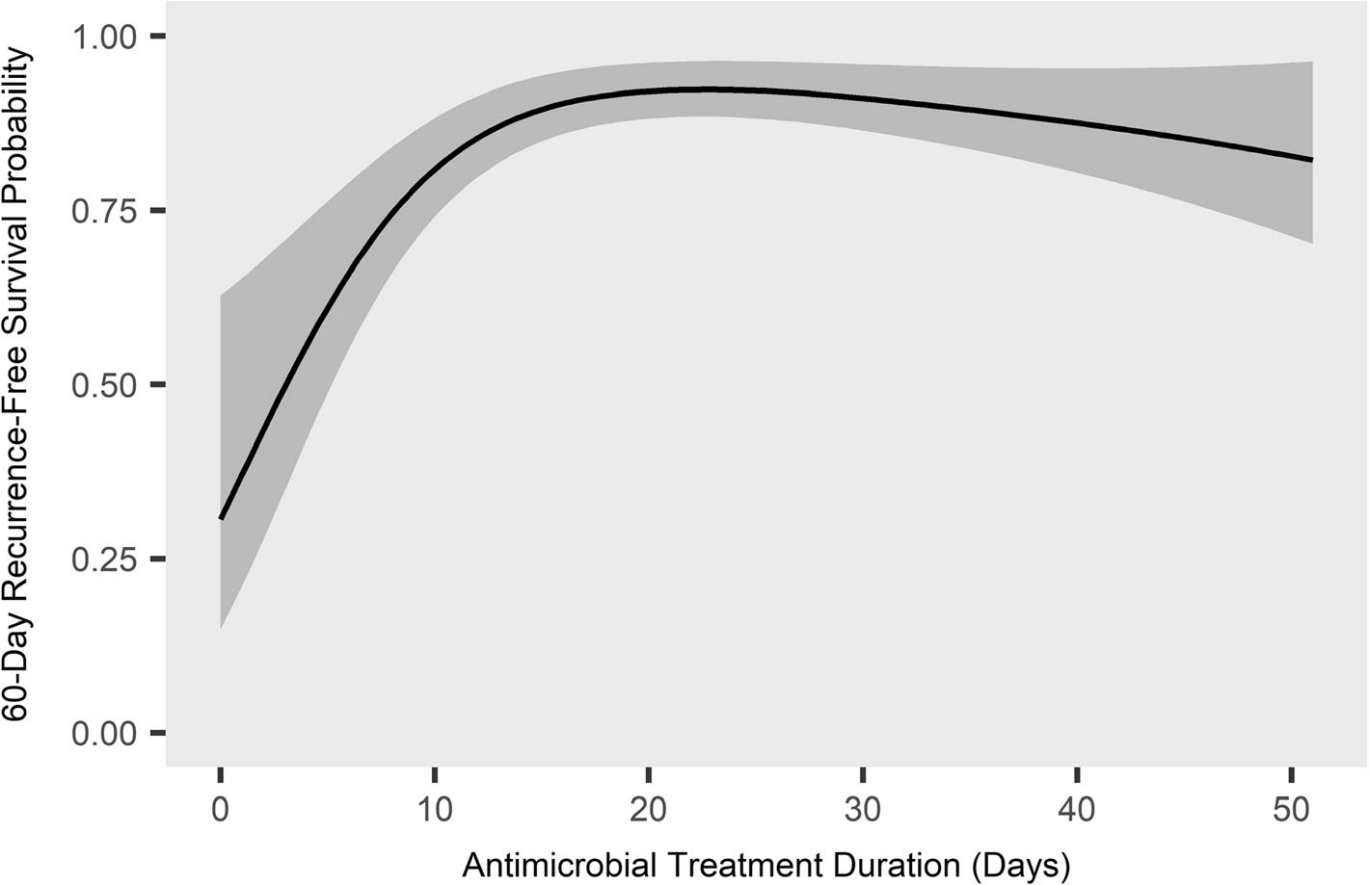
Predicted 60-day recurrence-free survival by antimicrobial treatment duration. The relationship between the number of days of effective antimicrobial treatment received and the predicted probability of 60-day recurrence-free survival is shown, based on the Cox proportional-hazards model described in Table 2. The shaded area indicates the 95% confidence interval. Other covariates in the model were adjusted to their median values: age 55 years, Sequential Organ Failure Assessment score 6, male sex, no immunosuppression, all central lines removed, and no high-risk organism present. For further details on individual variables, see Table 2

In the primary Cox model, antimicrobial treatment duration (hazard ratio (HR) = 0.35 for 14 days versus 7 days; 95% confidence interval (CI) 0.26–0.48), SOFA score (HR = 1.16 per point; 95% CI 1.09–1.22), and age (HR = 1.021 per year; 95% CI = 1.004–1.037) were associated with time to mortality or recurrence (Table 2). The association between antimicrobial treatment duration and time to mortality or recurrence appeared to plateau after approximately 15 days of effective antimicrobials (Fig. 2).

**Table 2:** Cox proportional-hazards model of risk factors for mortality or recurrence

Variables	Degrees of freedom	Hazard ratio^a^	95% confidence interval	*P* value
Age (per year)	1	1.021	1.004–1.037	0.01
Male	1	0.89	0.57–1.41	0.63
SOFA score (per point)	1	1.16	1.09–1.22	< 0.001
Immunosuppression present^b^	1	1.58	0.91–2.73	0.10
All central lines removed^c^	1	0.71	0.43–1.17	0.18
High-risk organism present^d^	1	1.33	0.81–2.17	0.26
Antimicrobial treatment duration^e^ (14 versus 7 days)	2	0.35	0.26–0.48	< 0.001

Age, SOFA score, and antimicrobial treatment duration were entered into the model as continuous variables; the remaining variables were entered as categorical variables

Non-linearity was allowed for antimicrobial treatment duration by means of restricted cubic splines with three knots

Linearity was assumed for other continuous variables

The outcome was time to mortality or recurrence, censored at 60 days after the completion of antimicrobial therapy

*SOFA* Sequential Organ Failure Assessment, *CLABSI* central line-associated bloodstream infection

^a^The hazard ratio for continuous variables compares each additional unit (e.g., the hazard ratio for age compares each additional year) except for antimicrobial treatment duration, for which it compares 14 days of antimicrobial treatment to 7 days

^b^Defined as absolute neutrophil count less than 500 cells/mm^3^, human immunodeficiency virus with a CD4 count less than 200 cells/mm^3^, or prior organ transplant on active immunosuppression

^c^Defined as all central lines removed within 4 days after CLABSI diagnosis

^d^Prespecified as *Staphylococcus aureus*, *Pseudomonas* species, and *Candida* species

^e^Defined as the continuous receipt of at least one antimicrobial to which the causative organism was sensitive, as determined by culture sensitivities; patients could miss no more than 1 consecutive day of antimicrobial therapy before treatment was considered complete unless they had therapeutic levels of an effective antimicrobial, as determined by monitoring of drug levels

Four separate sensitivity analyses excluding polymicrobial infections, *Staphylococcus aureus* infections, patients who received no effective antimicrobial therapy, and patients receiving fewer than 6 or greater than 16 days of antimicrobials showed a similarly significant association between shorter antimicrobial treatment duration and earlier mortality or recurrence (Additional file 1: Table S2). In a sensitivity analysis defining baseline time as the earlier of 14 days after initiation of antimicrobials or the completion of antimicrobial treatment, 91 of the 378 patients (24.1%) developed the primary outcome. Antimicrobial treatment duration was significantly associated with earlier mortality or recurrence in this model (HR = 0.41 for 14 days versus 7 days; 95% CI 0.31–0.55; Additional file 1: Table S2). A sensitivity analysis in which only antimicrobials administered after central line removal were considered effective antimicrobial treatment also showed a significant association between antimicrobial treatment duration and earlier mortality or recurrence (Additional file 1: Table S2).

A logistic regression model showed a significant association between antimicrobial treatment duration and 60-day mortality or recurrence (odds ratio (OR) = 0.39 for 14 versus 7 days; 95% CI 0.26–0.58; Additional file 1: Table S3). When the two components of the primary outcome (time to mortality and time to recurrence) were analyzed separately, shorter antimicrobial treatment was significantly associated with earlier mortality (HR = 0.35 for 14 versus 7 days; 95% CI 0.26–0.48) but not earlier recurrence (HR = 0.96 for 14 versus 7 days; 95% CI 0.75–1.25; Additional file 1: Table S3). When 60-day mortality and 60-day recurrence were analyzed separately in logistic regression models, similar results were obtained (Additional file 1: Table S3).

## Discussion

We found that increased age, increased SOFA score, and shorter antimicrobial treatment duration were all associated with earlier mortality or recurrence in patients with HA-CLABSIs. To our knowledge, this is the first study to examine factors associated with time to recurrence or mortality in patients with central line infections.

Current antimicrobial treatment duration guidelines for CLABSIs range from 5 to 7 days for uncomplicated coagulase-negative *Staphylococcus* infections in which the causative line is removed, to 6 weeks for complicated *Staphylococcus aureus* infections, with wide variation depending on patient and central line characteristics and causative organisms, although in most cases the guidelines recommend between 7 and 14 days of antimicrobial treatment [11]. We found an apparent plateauing of the benefit of prolonged antimicrobial treatment after about 15 days. We did not have enough patients to compare outcomes among individual classes of causative organisms, so this study should not, by itself, change current treatment guidelines. Instead, the significant association between shorter antimicrobial treatment duration and the primary outcome, which was robust and consistent in multiple sensitivity analyses, should be explored further in other, larger cohorts.

Guidelines recommend 2 to 6 weeks of antibiotics for most patients with *Staphylococcus aureus* CLABSIs, a longer course than that recommended for CLABSIs caused by other organisms [11]. A sensitivity analysis excluding *Staphylococcus aureus* CLABSIs did not change the association of antimicrobial treatment duration with the primary outcome, suggesting that *Staphylococcus aureus* was not the primary driver of this association. Moreover, the presence of high-risk organisms, which included *Staphylococcus aureus,* was not associated with the primary outcome.

While not specific to central line infections, Chotiprasitsakul et al. recently published a propensity score matched study of Enterobacteriaceae bacteremia with similar methodology to ours, finding no association between a longer (11–16 days) versus a shorter (6–10 days) duration of antibiotics and 30-day mortality [22]. Several differences between the studies may account for the disparate results. Chotiprasitsakul et al. limited their study to patients with Enterobacteriaceae bacteremia who received between 6 and 16 days of antibiotics, whereas ours included only patients with HA-CLABSIs, but with a broader range of causative organisms and with any antimicrobial treatment duration. Some organisms in our study, such as *Staphylococcus aureus*, may require more prolonged antimicrobial treatment. Although our study did not demonstrate this, it was underpowered to analyze organisms individually. Finally, our study analyzed antimicrobial duration as a continuous variable instead of categorizing it into long or short duration, increasing our power to detect an association with outcomes.

Time-to-event analysis was utilized in the primary analysis to increase the sensitivity of detecting a difference in outcomes as compared with logistic regression. While a longer time until mortality or recurrence may not be a patient-centered outcome, it was felt to be a surrogate for a difference in 60-day mortality or recurrence, as was suggested by the fact that the logistic regression analysis also demonstrated a significant improvement in outcomes with longer antimicrobial treatment. Furthermore, time-to-event analysis was performed in the previous study that was most similar to ours [22].

This study has several strengths. HA-CLABSI cases were independently and prospectively adjudicated by trained infection control staff in accordance with CDC guidelines and manually confirmed by the first author. Adjustments were made for multiple potentially confounding factors, including severity of illness and the potential for survivor bias. Variables for the Cox proportional-hazards model were selected a priori and the number of covariates was limited to prevent overfitting. Multiple sensitivity analyses were performed and were consistent with the primary analysis.

This study also has several limitations. As a retrospective, observational study in a single academic medical center, generalizability of results may be limited. The large percentage of immunosuppressed patients (37.2%) in particular may be different from other centers, although associations between immunosuppression and outcomes were not detected in the primary regression model. Despite our attempts to manage survivor bias, residual confounding may persist. Very early discontinuation of antimicrobials may be a surrogate measure for either quality or withdrawal of care, but a sensitivity analysis excluding patients who received fewer than 6 days of antimicrobials showed similar results as the primary analysis, suggesting that very early discontinuation of antimicrobials did not influence the results. Defining time zero as the end of antimicrobial treatment may introduce selection bias, as patients who survive 6 weeks of antimicrobials may be different from those who only survive to the end of a 2-week antimicrobial course; however, a sensitivity analysis using the earlier of 14 days after CLABSI diagnosis or the end of antimicrobial treatment as the baseline time still favored 14 over 7 days of antimicrobials. The CLABSI definition used in this study was developed for epidemiologic surveillance, so some CLABSIs in this study may not represent clinically significant infections. The CDC definition, however, is objective, has been used in prior CLABSI studies, and allows for independent adjudication of cases [4, 23]. Our antimicrobial treatment duration is a measure of total exposure to antimicrobials, whereas the IDSA CLABSI guidelines measure antimicrobial exposure starting from the first negative blood culture. While different definitions may influence the results, antimicrobial treatment duration remained significantly associated with time to mortality or recurrence in a sensitivity analysis using the date of central line removal as a surrogate for negative blood cultures. Estimation of PaO_2_ to FiO_2_ ratios from SpO_2_ to FiO_2_ ratios has only been validated in ventilated patients, who constituted a minority of patients in this study. Finally, due to the limited number of outcomes, some potentially important confounders, such as cirrhosis, end-stage renal disease, type and location of central line, and individual causative organism, were unable to be included in the primary regression model.

## Conclusions

In a retrospective cohort study of adult inpatients with hospital-acquired central line-associated bloodstream infections, longer duration of antimicrobial treatment was associated with longer recurrence-free survival. The improved outcomes associated with prolonged antimicrobial treatment appeared to plateau after the receipt of 15 days of antimicrobials.

## Additional file

Additional file 1: **Table S1.** Central line characteristics. Description of type, location, and insertion duration of central lines. **Table S2.** Sensitivity analyses performed and the results. **Table S3.** Analyses of secondary outcomes and their results. (DOCX 21 kb)

## Abbreviations

CDC: Centers for Disease Control and Prevention
CI: Confidence interval
CLABSI: Central line-associated bloodstream infection
FiO_2_: Fraction of inspired oxygen
HA-CLABSI: Hospital-acquired central line-associated bloodstream infection
HIV: Human immunodeficiency virus
HR: Hazard ratio
ICU: Intensive care unit
IDSA: Infectious Diseases Society of America
IQR: Interquartile range
NHSN: National Healthcare Safety Network
PaO_2_: Partial pressure of oxygen in arterial blood
SOFA: Sequential organ failure assessment
SpO_2_: Oxygen saturation

## References

[1] Centers for Disease Control and Prevention (2014). National and state healthcare-associated infections progress report.

[2] Warren DK, Quadir WW, Hollenbeak CS, Elward AM, Cox MJ, Fraser VJ (2006). Attributable cost of catheter-associated bloodstream infections among intensive care patients in a nonteaching hospital. Crit Care Med.

[3] Shannon RP, Patel B, Cummins D, Shannon AH, Ganguli G, Lu Y (2006). Economics of central line-associated bloodstream infections. Am J Med Qual.

[4] DiGiovine B, Chenoweth C, Watts C, Higgins M (1999). The attributable mortality and costs of primary nosocomial bloodstream infections in the intensive care unit. Am J Respir Crit Care Med.

[5] Ziegler MJ, Pellegrini DC, Safdar N (2014). Attributable mortality of central line associated bloodstream infection: systematic review and meta-analysis. Infection.

[6] Pittet D, Tarara D, Wenzel RP (1994). Nosocomial bloodstream infection in critically ill patients. Excess length of stay, extra costs, and attributable mortality. JAMA.

[7] Blot SI, Depuydt P, Annemans L, Benoit D, Hoste E, De Waele JJ (2005). Clinical and economic outcomes in critically ill patients with nosocomial catheter-related bloodstream infections. Clin Infect Dis.

[8] Fowler VG, Justice A, Moore C, Benjamin DK, Woods CW, Campbell S (2005). Risk factors for hematogenous complications of intravascular catheter-associated Staphylococcus aureus bacteremia. Clin Infect Dis.

[9] Corey GR, Stryjewski ME, Everts RJ (2009). Short-course therapy for bloodstream infections in immunocompetent adults. Int J Antimicrob Agents.

[10] Havey TC, Fowler RA, Daneman N (2011). Duration of antibiotic therapy for bacteremia: a systematic review and meta-analysis. Crit Care.

[11] Mermel LA, Allon M, Bouza E, Craven DE, Flynn P, O’Grady NP (2009). Clinical practice guidelines for the diagnosis and management of intravascular catheter-related infection: 2009 update by the Infectious Diseases Society of America. Clin Infect Dis.

[12] Malanoski GJ, Samore MH, Pefanis A, Karchmer AW (1995). Staphylococcus aureus catheter-associated bacteremia. Minimal effective therapy and unusual infectious complications associated with arterial sheath catheters. Arch Intern Med.

[13] Chong YP, Moon SM, Bang KM, Park HJ, Park SY, Kim MN (2013). Treatment duration for uncomplicated staphylococcus aureus bacteremia to prevent relapse: analysis of a prospective observational cohort study. Antimicrob Agents Chemother.

[14] Huerta LE, Nelson GE, Rice TW. The effect of antimicrobial treatment duration on mortality and recurrence in hospital-acquired central line-associated bloodstream infections. In: D24 critical care: the other half of the ICU—update in management of non-pulmonary critical care: American Thoracic Society; 2017. p. A7148. Available at: https://www.atsjournals.org/doi/abs/10.1164/ajrccm-conference.2017.195.1_MeetingAbstracts.A7148.

[15] Centers for Disease Control and Prevention. National Healthcare Safety Network ( NHSN ) patient safety component manual. https://www.cdc.gov/nhsn/pdfs/pscmanual/pcsmanual_current.pdf. Accessed 26 Apr 2017.

[16] Vincent JL, Moreno R, Takala J, Willatts S, De Mendonça A, Bruining H (1996). The SOFA (Sepsis-related Organ Failure Assessment) score to describe organ dysfunction/failure. Intensive Care Med.

[17] Brown SM, Duggal A, Hou PC, Tidswell M, Khan A, Exline M (2017). Nonlinear imputation of PaO2/FIO2 from SpO2/FIO2 among mechanically ventilated patients in the ICU. Crit Care Med.

[18] Yu S, Leung S, Heo M, Soto GJ, Shah RT, Gunda S (2014). Comparison of risk prediction scoring systems for ward patients: a retrospective nested case-control study. Crit Care.

[19] Concato J, Peduzzi P, Holford TR, Feinstein AR (1995). Importance of events per independent variable in proportional hazards analysis I. Background, goals, and general strategy. J Clin Epidemiol.

[20] Peduzzi P, Concato J, Feinstein AR, Holford TR (1995). Importance of events per independent variable in proportional hazards regression analysis II. Accuracy and precision of regression estimates. J Clin Epidemiol.

[21] Peduzzi P, Concato J, Kemper E, Holford TR, Feinstein AR (1996). A simulation study of the number of events per variable in logistic regression analysis. J Clin Epidemiol.

[22] Chotiprasitsakul D, Han JH, Cosgrove SE, Harris AD, Lautenbach E, Conley AT (2018). Comparing the outcomes of adults with Enterobacteriaceae bacteremia receiving short-course versus prolonged-course antibiotic therapy in a multicenter, propensity score-matched cohort. Clin Infect Dis.

[23] Yokota PKO, Marra AR, Belluci TR, Victor E da S, dos SOFP, Edmond MB (2016). Outcomes and predictive factors associated with adequacy of antimicrobial therapy in patients with central line-associated bloodstream infection. Front Public Heal.

