# Supplementary material for: Factors associated with recurrence and mortality in central line-associated bloodstream infections: a retrospective cohort study
Source: Crit Care. 2018 Oct 26;22:266. doi: 10.1186/s13054-018-2206-7 (PMC6204025; doi:10.1186/s13054-018-2206-7)
Supplement: Supplementary file 1 — Table S1. Central line characteristics. Description of type, location, and insertion duration of central lines. Table S2. Sensitivity analyses performed and the results. Table S3. Analyses of secondary outcomes and their results. (DOCX 21 kb) [file 13054_2018_2206_MOESM1_ESM.docx]

Table S1: Central Line Characteristics

| Characteristics | Complete Cases  (N = 366) | Patients with 60-Day Mortality or Recurrence  (N = 81) | Patients with 60-Day Recurrence-Free Survival  (N = 285) | P-value^a^ |
| --- | --- | --- | --- | --- |
| Central Line Type |  |  |  |  |
| Temporary Central Venous Catheter^b^ | 128 (35.0) | 30 (37.0) | 98 (34.4) | 0.66 |
| PICC | 180 (49.2) | 41 (50.6) | 139 (48.8) | 0.77 |
| Tunneled Central Line | 46 (12.6) | 11 (13.6) | 35 (12.3) | 0.76 |
| Implanted Port | 41 (11.2) | 7 (8.6) | 34 (11.9) | 0.41 |
| Insertion Location |  |  |  |  |
| Internal Jugular | 124 (33.9) | 28 (34.6) | 96 (33.7) | 0.88 |
| Subclavian | 79 (21.6) | 16 (19.8) | 63 (22.1) | 0.65 |
| Femoral | 22 (6.0) | 9 (11.1) | 13 (4.6) | 0.04 |
| Peripheral Vein | 173 (47.2) | 40 (49.4) | 133 (46.7) | 0.67 |
| Multiple Central Lines^c^ | 37 (10.1) | 13 (16.0) | 24 (8.4) | 0.04 |
| Inpatient Days with Causative Central Line in Place Prior to CLABSI Diagnosis | 8 [4-14] | 7 [4-14] | 8 [4-14] | 0.28 |
| PICC = Peripherally inserted central catheter; CLABSI = Central-line associated bloodstream infection  Values are presented as number (percentage) or median [interquartile range] as appropriate  Central lines at our institution are inserted using sterile technique and a pre-procedure time-out and checklist, including maximal barrier precautions (sterile gown and gloves, cap, mask, full-body drape), skin antisepsis with chlorhexidine, and antimicrobial-impregnated dressings. A standardized institutional nursing protocol is used when accessing central lines. It mandates hand hygiene before and after manipulating the line and aseptic technique when accessing the catheter hub or injection caps. When accessing the line, staff must scrub the access port with an alcohol or chlorhexidine pad using a twisting motion 5 times around the threads and scrub 5 times across the septum, letting it dry before accessing. Lumens must remain capped and protected at all times, and a sterile, needleless connector applied to a lumen if not in use. If a needleless connector is removed for a continuous infusion, the lumen/cap interface must be cleaned and allowed to dry before removal  ^a^Univariate analyses were performed with Mann-Whitney U tests for continuous variables and Chi-square or Fisher’s exact tests for categorical variables  ^b^Includes temporary single and multi-lumen catheters  ^c^Thirty-seven patients had 2 central lines in place at the time of CLABSI diagnosis for a total of 403 central lines. No patient had more than 2 central lines in place at the time of diagnosis. | | | | |

Table S2: Sensitivity Analyses

| Analysis | Variables^a^ | Outcomes^b^ | Number of Patients (Number of Events) | Hazard Ratio for Antimicrobial Treatment Duration^c^ | 95% Confidence Interval | P-value |
| --- | --- | --- | --- | --- | --- | --- |
| Cox proportional-hazards model excluding polymicrobial infections | Same as primary analysis^d^ | Time to mortality or recurrence | 317 (71) | 0.36 | 0.26-0.49 | < 0.001 |
| Cox proportional-hazards model excluding *Staphylococcus aureus* infections | Same as primary analysis | Time to mortality or recurrence | 317 (71) | 0.31 | 0.22-0.44 | < 0.001 |
| Cox proportional-hazards model excluding patients receiving no effective antimicrobial treatment | Same as primary analysis | Time to mortality or recurrence | 350 (70) | 0.30 | 0.20-0.45 | < 0.001 |
| Cox proportional-hazards model excluding patients receiving fewer than 6 or greater than 16 days of antimicrobials | Age (years), SOFA score, antimicrobial treatment duration^e^ (days) | Time to mortality or recurrence | 189 (33) | 0.43 | 0.20-0.91 | 0.03 |
| Cox proportional-hazards model in patients with all central lines removed prior to the completion of antimicrobial treatment defining the start of antimicrobial treatment as the date of final central line removal | Age (years), SOFA score, antimicrobial treatment duration (days), high-risk organism present^f^ | Time to mortality or recurrence | 285 (49) | 0.39 | 0.25-0.61 | < 0.001 |
| Cox proportional-hazards model defining baseline time as the earlier of 14 days after initiation of antimicrobials or the end of antimicrobial treatment | Same as primary analysis | Time to mortality or recurrence | 378 (91) | 0.41 | 0.31-0.55 | < 0.001 |
| SOFA = Sequential Organ Failure Assessment  ^a^Age, SOFA score, and antimicrobial treatment duration were entered as continuous variables in all analyses in which they were included. All other variables were entered as categorical variables. Linearity was assumed for all continuous variables unless otherwise stated.  ^b^Outcomes were censored 60 days after completion of antimicrobial therapy.  ^c^Hazard ratios and odds ratios for antimicrobial treatment duration compared 14 days of treatment to 7 days.  ^d^Variables included in the primary analysis were: age (years), SOFA score, antimicrobial treatment duration (days) as a restricted cubic spline with 3 knots, immunosuppression, all central lines removed, high-risk organism present, and male gender. For more details on the primary analysis, see Table 2.  ^e^Defined as the continuous receipt of at least one antimicrobial to which the causative organism was sensitive, as determined by culture sensitivities. Patients could miss no more than one consecutive day of antimicrobial therapy before treatment was considered complete unless they had therapeutic levels of an effective antimicrobial, as determined by monitoring of drug.  ^f^Prespecified as *Staphylococcus aureus*, *Pseudomonas* species, and *Candida* species. | | | | | | |

Table S3: Analyses of Secondary Outcomes

| Analysis | Variables^a^ | Outcomes^b^ | Hazard Ratio/ Odds Ratio for Antimicrobial Treatment Duration^c^ | 95% Confidence Interval | P-value |
| --- | --- | --- | --- | --- | --- |
| Cox proportional-hazards model for time to 60-day mortality | Age (years), SOFA score, antimicrobial treatment duration^d^ (days) as restricted cubic spline with 3 knots, all central lines removed^e^, high-risk organism present^f^ | Time to mortality | 0.35 | 0.26-0.48 | < 0.001 |
| Cox proportional-hazards model for time to 60-day recurrence | Age (years), SOFA score, antimicrobial treatment duration (days) | Time to recurrence | 0.96 | 0.75-1.25 | 0.79 |
| Logistic regression model for 60-day mortality or recurrence | Age (years), SOFA score, antimicrobial treatment duration (days) as restricted cubic spline with 3 knots, male sex, all central lines removed, high-risk organism present, immunosuppression present | 60-day mortality or recurrence | 0.39 | 0.26-0.58 | < 0.001 |
| Logistic regression model for 60-day mortality | Age (years), SOFA score, antimicrobial treatment duration (days) as restricted cubic spline with 3 knots, all central lines removed, high-risk organism present | 60-day mortality | 0.36 | 0.24-0.53 | < 0.001 |
| Logistic regression model for 60-day recurrence | Age (years), SOFA score, antimicrobial treatment duration (days) | 60-day recurrence | 1.02 | 0.81-1.28 | 0.88 |
| SOFA = Sequential Organ Failure Assessment; CLABSI = central line-associated bloodstream infection  ^a^Age, SOFA score, and antimicrobial treatment duration were entered as continuous variables in all analyses in which they were included. All other variables were entered as categorical variables. Linearity was assumed for all continuous variables unless otherwise stated.  ^b^Outcomes were censored 60 days after completion of antimicrobial therapy. Number of deaths within 60 days = 62. Number of recurrences within 60 days = 26.  ^c^Hazard ratios and odds ratios for antimicrobial treatment duration compared 14 days of treatment to 7 days.  ^d^Defined as the continuous receipt of at least one antimicrobial to which the causative organism was sensitive, as determined by culture sensitivities. Patients could miss no more than one consecutive day of antimicrobial therapy before treatment was considered complete unless they had therapeutic levels of an effective antimicrobial, as determined by monitoring of drug.  ^e^Defined as all central lines removed within 4 days of CLABSI diagnosis.  ^f^Prespecified as *Staphylococcus aureus*, *Pseudomonas* species, and *Candida* species. | | | | | |
